# A Novel Interaction between Chemokine and Phosphoinositide Signaling in Metastatic Prostate Cancer

**DOI:** 10.18103/mra.v11i7.1.4020

**Published:** 2023-07-06

**Authors:** Codrut Radoiu, Barani Govindarajan, Michael Wang, Diego Sbrissa, Michael L. Cher, Sreenivasa R. Chinni

**Affiliations:** 1 Department of Urology, Wayne State University School of Medicine, Detroit, MI 48201, USA.; 2 Department of Pathology, Wayne State University School of Medicine, Detroit, MI 48201, USA.; 3 Department of Oncology, Wayne State University School of Medicine, Detroit, MI 48201, USA.

## Abstract

Prostate cancer commonly metastasizes to bone due to its favorable microenvironment for cell growth and survival. Currently, the standard of care for metastatic prostate cancer is medical castration in conjunction with chemotherapeutic agents and newer anti-androgen/androgen receptor therapies. While these therapies aim to improve the quality of life in patients with advanced disease, resistance to these therapies is inevitable prompting the development of newer therapies to contain disease progression. The CXCL12/CXCR4 axis has previously been shown to be involved in prostate cancer cell homing to bone tissue, and new investigations found a novel interaction of Phosphatidyl Inositol 4 kinase IIIa (PI4KA) downstream of chemokine signaling. PI4KA phosphorylates at the 4th position on phosphatidylinositol (PI), to produce PI4P and is localized to the plasma membrane (PM). At the PM, PI4KA provides precursors for the generation of PI(4,5)P_2,_ and PI(3,4,5)P_3_ and helps maintain PM identity through the recruitment of lipids and signaling proteins. PI4KA is recruited to the PM through evolutionarily conserved adaptor proteins, and in PC cells, CXCR4 binds with adaptor proteins to recruit PI4KA to the PM. The objective of this review is to summarize our understanding of the role that phosphatidyl inositol lipid messengers in cancer cells.

## Current Strategies for treatment of prostate cancer with emphasis on metastatic disease

Prostate cancer is the most commonly diagnosed cancer and is the second most common cause of cancer-related death in American men^1^. Recent studies have demonstrated a global decline in prostate cancer incidence in men; however, the number of metastatic hormone-sensitive prostate cancer (mHSPC) continues to increase^2^. Common metastatic sites include lymph nodes, bones, liver, and lungs. Those with high-volume disease, commonly defined as four or more bony metastasis (with one outside of the pelvis/spinal column) and/or visceral metastasis, often have a worse prognosis due to the extent of the distant cancerous spread^3^. The progression of this cancer is associated with debilitating symptoms, including bone pain that results from invasion of the skeleton, resulting in fractures that can affect mobility, disrupting activities of daily living^4^. The management of advanced prostate cancer was revolutionized with the use of androgen deprivation therapy (ADT) and has evolved rapidly over the past 10 years with the use of cytotoxic agents and novel androgen signaling inhibitors (ASI). The objective of this summary will be to investigate the advances made in the different modalities to manage metastatic prostate cancer.

mHSPC is defined as metastatic prostate cancer that responds to ADT, which is the backbone of all metastatic prostate cancer therapy. Castration can be achieved surgically through bilateral orchiectomy, or medically via parenteral administration of luteinizing hormone-releasing hormone (LHRH) agonists or antagonists. LHRH antagonists such as degarelix, or LHRH agonists, such as leuprolide, are commonly used to create medical castration^4,5^. Health-related quality of life in men undergoing ADT is significantly affected due to hot flashes, sexual, urinary, and bowel dysfunction, and an increased risk in depression^4^. No differences have been observed in long-term quality of life between men receiving LHRH agonists vs antagonists concerning the domains highlighted earlier. Relugolix, a newer orally-administered LHRH antagonist, has demonstrated an improved safety profile regarding cardiovascular health and may be more effective at testosterone suppression compared to LHRH agonist therapy^6^.

The first couplet therapy for mHSPC is a chemotherapy agent, docetaxel, from the landmark studies of CHAARTED, STAMPEDE, and GETUG-15^4,7,8^. Docetaxel + ADT was the first drug to demonstrate an improvement in overall survival (OS) compared to ADT alone for those with mHSPC. Common side effects of hair loss, neuropathy, and neutropenia were noted with docetaxel treatment. Soon after, multiple landmark studies confirmed the significant OS advantage with the addition of ASIs, such as abiraterone, apalutamide, enzalutamide, in addition to ADT, compared to monotherapy of ADT^9–12^. As a result, couplet therapy has become the standard of care for patients with mHSPC. Abiraterone is an inhibitor of extragonadal testosterone production by inhibiting 17 alpha-hydrolase and 17,20-lyase and has the side effects of hypertension and hypokalemia^13^. Apalutamide and enzalutamide are androgen signaling receptor inhibitors with side effects that include hypertension and memory impairment^14^. All three of these drugs coupled with ADT have been shown to increase overall survival compared to ADT alone for both high volume and low volume mHSPC. In contrast, docetaxel + ADT compared to ADT alone did not show statistical significance for OS for those with low volume mHSPC in a subgroup analysis of CHAARTED and STAMPEDE trials, changing the treatment paradigm for patients with high vs low volume mHSPC^8^.

Over the past year, two large trials examining triplet therapy was recently published – PEACE-1 trial with ADT + docetaxel + abiraterone, and ARASENS trial with ADT + docetaxel + darolutamide^15,16^. In both of these trials, patients with high volume mHSPC had improved OS with the triplet therapy compared to the couplet therapy of ADT + docetaxel. The data for low volume mHSPC is less clear. As a result, men with low volume, low grade mHSPC may benefit more with couplet therapy with ASI than with triplet therapy due to fewer side effects, though more studies need to be conducted in this space.

If PSA continues to rise or if there is a progression of disease radiographically despite castration, then the patient is deemed to have castrate resistance prostate cancer (CRPC). This is often due to persistent androgen production by tumor sites, androgen receptor deregulation, and aberrant modifications, despite castration^17^. Many of the same drugs discussed before such as abiraterone, enzalutamide, and docetaxel are also relevant in the setting of metastatic CRPC (mCRPC). Other agents like Sipleucel-T may be useful scenarios where patients are minimally symptomatic without visceral metastasis^18^. Radium-223 is useful for patients with symptomatic bone lesions without visceral metastases by emitting alpha particles targeted towards the bony metastases, achieving the goal of depleting bony metastasis in nearby areas^19^. Newer agents, such as lutetium-177-prostate-specific membrane antigen (PSMA)-617 are being used in patients previously treated with an ARSI and a taxane based therapy with PSMA positive metastases, and has been shown to improve OS and progression free survival^20^. Moreover, with the utilization of germline and somatic testing, immunotherapy and targeted therapy drugs like pembrolizumab, olaparib, and rucaparib have been demonstrated with favorable outcome for patients with specific mutations^21–23^.

Metastatic prostate cancer is discovered late in the disease’s progression and is associated with a drastic decline in quality of life due to physical, emotional, and psychological impairments. Management with ADT, ARSI, novel chemotherapy, immunotherapy, and targeted therapy improves long-term quality of life and survival in patients with advanced mHSPC and mCRPC. Innovation regarding therapies targeting and inhibiting metastasis is sought after for future advancements in the management of patients with prostate cancer to prevent the complications highlighted in this summary.

## The role of chemokine CXCL12 and its receptor CXCR4 in bone metastasis.

The propensity of prostate cancer to metastasize to bone has long been investigated, with a great focus being placed on the mechanisms controlling tumor cell homing to the bone and subsequent interaction of tumor cells with various components of the bone microenvironment. Approximately 70% of prostate cancer metastasis initially targets bone, resulting in further complications, including predominantly osteoblastic lesions. Even though the lesions are often osteoblastic, these lesions also contain osteolytic activity. Tumor cells in bone secrete several factors which promote the recruitment, differentiation, and activity of osteoblasts and osteoclasts, leading to bone degradation and formation. Tumor cell-induced bone degradation through osteoclast activity release several latent growth factors from the bone matrix and aid in tumor cell growth and proliferation, thus, creating a vicious cycle supporting bone tumor growth.

Tumor cells homing to the bone marrow mimics the dynamics of hematopoietic stem cell homing, where initially interact with bone marrow endothelium through cell surface adhesion molecules and subsequently get into bone marrow. Chemokine CXCL12 and its receptor CXCR4 has been shown to facilitate HSC homing to the bone. Osteoblasts in bone marrow express CXCL12, promoting prostate cancer cell invasion of the niche by chemokine signaling by CXCR4 on the invading cells. Once inside the bone marrow environment, the prostate cancer cells compete with the hematopoietic cells for occupancy in the bone marrow. The involvement of the CXCR12/CXCR4 axis in prostate cancer (PC) bone metastasis has been a promising research focus. Expression of CXCR4 is under regulation by the ERG transcription factor, which is typically overexpressed in prostate tumors with a TMPRSS2-ERG gene fusion^24^. Upon androgen induction of the TMPRSS2 gene promoter in PC cells, overexpression of ERG due to the aberrant gene fusion consequently increases levels of CXCR4^24,25^. Elevated CXCR4 in patients has been found to be associated with poorer survival and prognosis as compared to others with lower CXCR4 expression^26^. The loss of tumor suppressor phosphatase and tensin homolog (PTEN) is associated with PC progression and is one of the most commonly lost tumor suppressor genes in prostate cancers^27^. The PTEN loss in PC cells is associated with enhanced expression of CXCL12 and CXCR4 expression and contributes to tumor growth and metastasis^28^. This receptor localizes to lipid rafts and exerts its function in cellular invasion upon binding of its ligand, CXCL12. CXCL12/CXCR4 signaling in prostate cancer cells has been shown to express several members of soluble and membrane-type metalloproteinases, MMP-1, MMP-2, MMP-3, MMP-9, MMP-10, MMP-11, and MMP-14 whose expression contribute to cellular migration and invasion^29–31^.

Newly arrived cancer cells in the bone microenvironment compete with the endosteal hematopoietic stem cell (HSC) niche for the initial establishment of bone metastasis. Plerixafor is a CXCR4 antagonist currently used in clinics for HSC mobilization from bone marrow by disrupting HSC niche interaction with the bone microenvironment. Treating mice with plerixafor leads to disrupting the occupancy of PC cells at the HSC niche^32^ and inhibits the initial establishment of metastases, which leads to inhibition of bone tumor growth^32,33^. The subsequent outgrowth of the bone tumors is sensitive to EGFR inhibition suggesting that alternate pathways contribute to the expansion of osseous metastases^33^. To identify the novel interacting proteins contributing to the CXCR4-mediated bone tumor growth, we developed a stable model of PC3 prostate cancer cells with knockdown and overexpressing CXCR4. SILAC-based quantitative proteomic analysis of lipid raft microdomains identified phosphatidylinositol 4-kinase III-alpha (PI4KA) as a novel downstream modulator of CXCL12/CXCR4 signaling in prostate cancer cells^34^.

## Novel role of phosphatidylinositol 4-kinase III-alpha (PI4KA) in prostate cancer cells:

**Figure 1.**
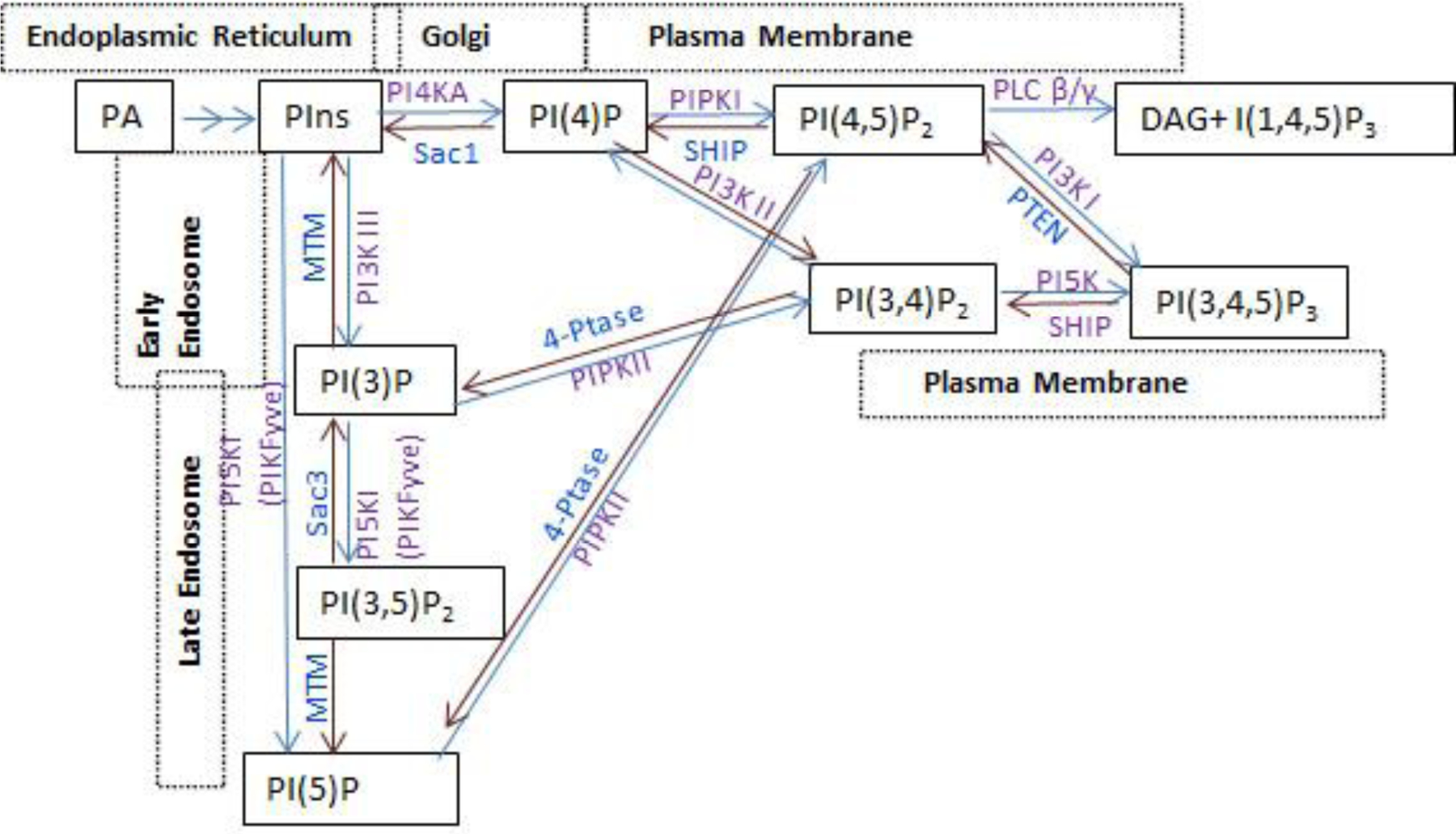
Metabolism of the phosphoinositides and their subcellular localization.

Phosphoinositides represent a minor component of the eukaryotic cell membranes and are involved in key functions of cell physiology^35,36^. They are formed by phosphorylation of the inositol ring at the 3,4,5 position of phosphatidylinositol (PI), resulting in seven phosphoinositide species (Fig. 1). The seven main phosphoinositide species are PI4P, PI3P, PI5P, PI(4,5)P_2_, PI(3,5)P_2_, PI(3,4)P_2_ and PI(3,4,5)P_3_. A network of interconverting enzymes regulates phosphorylation and dephosphorylation PIs resulting in the fine spatiotemporal regulation of PI species in cells. The head group of the phosphoinositol ring recruits the effector proteins at the membrane-cytosol interface to control various cellular activities such as membrane budding and fusion, vesicular transport, actin, and microtubule assembly. The focus of this review is to emphasize the alterations in PI metabolism in cancer progression, as this aspect of normal cellular physiology is covered by previous studies^35,37^. Many enzymes regulating phosphoinositide species are often dysregulated in cancers due to overexpression, mutations leading to altered activities, and loss of gene expression. To determine the changes in the cellular phosphoinositide species, we performed quantitation of seven different PI species in cells using HPLC based on our previously established method^38,39^. A non-tumorigenic prostate epithelial cell line, CRL2221, and two tumorigenic cell lines derived from soft tissue metastasis, LNCaP, and bone metastasis, VCaP, were analyzed for determining PI profiles (Table). PI4P is the most abundant PI in cells, whereas its levels were decreased in prostate tumor cells, and a concomitant increase in all three species of PIP2 (PI(4,5)P_2_, PI(3,5)P_2_, PI(3,4)P_2_). In prostate tumors, PI4KA is overexpressed in metastasis ^34^ and the higher levels of PIP2 in cancer cells is due high activity of PI4KA and efficient subsequent conversion of PI4P to PI(4,5)P_2_, and PI(3,4)P_2._ PI(4,5)P_2_ is the second abundant PtdIns species in cells, and its levels increased 4 to 5 folds in tumor cells. PI(3,4,5)P_3_ levels were increased from 9 to 12 folds in tumor cells. Among the PIs, PI4P, PI(4,5)P_2,_ and PI(3,4,5)P_3_ were highly localized to the plasma membrane and potent signal transducers in all cells, and all these three PIs are highly expressed in prostate metastatic tumor cells. PI4KA is the enzyme responsible for the production of PI4P in the plasma membrane. PI4KA is recruited to the PM by an assembly of adaptor proteins - Eighty-five Requiring 3 (EFR3) (a palmitoylated scaffold protein), Tetratricopeptide repeat protein (TTC7), and FAM126. Two heterotrimeric complexes of PI4KA-TTC7-FAM126 dimerize with each of their C-terminal portions, forming a hexametric complex, with TTC7-FAM126 directly interacting with the c-terminus of EFR3 through electrostatic interactions and recruited to the PM^40–42^. An alternative targeting of PI4KA is proposed where PI4KA and EFR3 interact with an integral PM protein TMEM150^43^. In prostate cancers, PI4KA is overexpressed in metastasis and several chemokine receptors are shown to interact with EFR3 and TTC7 suggesting this interaction brings PI4KA to the PM upon chemokine signaling and contributing to the prostate cancer cell invasion and metastasis^34^. EFR3A-PI4KA interaction on the PM has been implicated in many KRAS-dependent tumors, and treatments with PI4KA inhibitors along with KRAS inhibitors, or MAPK and PI3K inhibitors have proved to improve efficacy in cancer cell growth inhibition^44^. This lipid kinase complex comprising adaptor proteins is conserved from yeast to humans^40^.

The main function of PI4P in PM is to maintain stable PI(4,5)P_2_ levels, which is used by the phospholipase C (PLC) enzyme to break down and produce two key intracellular second messengers, inositol 1, 4, 5-triphosphate (Ins(1,4,5)P_3_) and diacylglycerol (DAG). PI4KA controls the PM levels of PI(4,5)P_2_ by replenishing it when it is utilized by PLC in PM and this activity is mediated by both PM and Golgi-associated PI4KA^42,45–47^. PM-produced PI4P also contributes to the Phosphatidyl serine transport from the ER to the inner lipid bilayer of the PM against the natural gradient at the ER-PM junction to maintain PM lipid composition. This non-vesicular lipid transport is mediated by members of oxysterol binding protein (OSBP)-related protein (ORP) with its PH domain binding PI4P. The ER-associated lipid phosphatase Sac1 dephosphorylate PI4P and, thus, the combined activity of PI4KA and Sac1 maintains the PI4P gradient across PM and ER^28^. PM-generated PI4P also regulates the activity of store-operated calcium entry by interacting with regulatory protein Stim1 through its polybasic binding domain, thus regulating calcium entry into the cells^48^. The accumulated PI4P inner leaflet of PM contributes to negative charges interacting with proteins containing polybasic binding domains. PI4P produced in Golgi by the action of PI4KB, PI4K2A, and PI4K2B has also been shown to promote enhanced secretory phenotype through overexpression in several types of cancers and PI4P binding protein GOLPH3 has been shown to mediate oncogenic function through promoting cell motility, matrix interaction and invasion leading to the progression of metastasis^49^.

PI(4,5)P_2_ production from the PI4P in PM is mediated by the PI4P5K enzymes, in cancer members of PIP5K are shown to regulate cell migration and invasion through its known function on invadopodium formation, where PI(4,5)P_2_ binding to talin regulate integrin induced extracellular matrix adhesion of cancer cells^27,41,50–54^. The overexpression and constitutive activation of PI4P5K convert PM-produced PI4P to PI(4,5)P_2_, which regulates actin-associated cytoskeletal proteins^55^ contributing to tumor cell motility. In addition, a direct metabolic channeling of PI4P for the production of PI(4,5)P_2_, and PI(3,4,5)P_3_ has been reported through interaction of PI4KA, PI4P5K and PI3K enzymes with a scaffold protein IQ-motif containing GTPase activating protein 1 (IQGAP1)^51^. Thus, this channeling process would convert the PI4P to PI(4,5)P_2_ and this concept supports our observation that in metastatic cells the PI(4,5)P_2_ and PI(3,4,5)P_3_ levels are high compared to non-tumorigenic cells. Whether this direct interaction between kinases mediating the high levels of PtdIns conversion needs to be verified in prostate cancer cells.

PI(3,4,5)P_3_ is a minor PtdIns species present in the inner leaflet of PM. Activated class 1 PI3K mediates the rapid generation of PI(3,4,5)P_3_ from PI(4,5)P_2_ in PM without significantly altering the levels of PI(4,5)P_2_ and a key function of this lipid to recruit plexin homology (PH) domain effector proteins to PM. Akt is a key downstream signaling mediator of PI(3,4,5)P_3_ mediates cell proliferation, growth, and survival. In CRPC-activated PI3K crosstalk with AR leads to the growth of prostate cancers at low androgen levels in tumors suggesting the significance of PIP3 and its downstream signaling pathways on the lethal progression of PC. Thus, sequential synthesis of PI4P - PI(4,5)P_2_-PI(3,4,5)P_3_ contributes to coordinated control of signal transduction in tumor cells and promotes tumor growth.

## Conclusions:

Androgen deprivation therapies along with androgen signaling inhibitors most likely extinguish the AR signaling in metastatic hormone sensitive prostate cancers and often these therapies lead to the development of castrate resistant metastatic disease. In-depth understanding of the resistance mechanisms would facilitate the development of newer therapies for this phase of the disease. CXCL12/CXCR4 signaling has been implicated in the development of metastatic disease in PC and anti-chemokine therapies limit the initial establishment of bone metastases in mice models through targeting tumor stromal interactions in bone tumor microenvironment.

Lipid kinase PI4KA identified as a novel target downstream of CXCR4 in prostate cancer cells and in PC cells chemokine signaling induces PI4KA activation for PI4P production at PM. In PM PI4P can serve as a precursor for more potent PIs for cellular signaling and maintain lipid homeostasis. Future research will show molecular mechanism how PI4KA is recruited to the PM and its impact on PC progression by elucidating the molecular mechanisms contributing to cell migration and invasion. Deeper understanding of PI4KA activation could provide clues for developing newer therapies for targeting the PI signaling in cancer cells. Analyzing the function of the downstream phosphoinositide signaling pathways and their impact on regulating cellular growth and survival can aid in narrowing the target for developing novel anti-cancer agents. Further, horizontal targeting of key enzymes regulating the PtdIns pathway (PI4P-PI(4,5)P_2_-PI(3,4,5)P_3_) with multiple inhibitors may constitute novel strategies for prostate cancer treatment.

## Figures and Tables

**Table.** Quantitation of cellular PtdIns species in prostate epithelial and cancer cells.

Cellular metabolite profiles of different Ptdlns species (% of total species)							
Cells	PI3P	PI4P	PI5P	PI3,5P_2_	PI3,4P_2_	PI4,5P_2_	PI3,4,5P_3_
**CRL2221**	**1.41**	**92.3**	**4.06**	**0.012**	**0.020**	**2.19**	**0.041**
**LNCaP**	**1.15**	**80.4**	**5.85**	**2.38**	**0.55**	**11.4**	**0.39**
**VCaP**	**2.10**	**79.8**	**7.53**	**1.15**	**0.35**	**8.60**	**0.51**
